# Social Unrest and Its Implications on the Pediatric Dermatological Patient: Lessons From a Single Center Academic Dermatology Department

**DOI:** 10.1111/pde.70260

**Published:** 2026-06-14

**Authors:** Colby L. Presley, John Meisenheimer, Chandler W. Rundle, Sheilagh Maguiness, Ian A. Maher, Alyce J. M. Anderson

**Affiliations:** ^1^ Department of Dermatology University of Minnesota Minneapolis Minnesota USA

**Keywords:** clinical operations, pediatric dermatology, political unrest, social unrest, teledermatology

## Abstract

The medical care of the pediatric population is disproportionately affected compared to adult medical care during periods of social and political unrest. No‐show and late cancellation rates (NSLCR) were collected across pediatric and adult dermatology clinics in Minneapolis, Minnesota during recent periods of social unrest and the year prior. Pediatric dermatologic appointments, specifically those in metropolitan areas, were found to have a statistically significant increase (*p* < 0.001) in NSLCR compared to adult dermatology clinics.

Political and social unrest can result in medical care disruption [1]. Unfortunately, children are at the highest risk of being disproportionately negatively impacted during civil turbulence [2]. Minneapolis, Minnesota, is no stranger to social unrest. Immigration and Customs Enforcement (ICE) initiated Operation Metro Surge, increasing its presence within the Minneapolis metropolitan area. After the local deaths of Renee Good and our healthcare colleague, Alex Pretti, tensions have soared as we navigate serving our diverse populations during this unprecedented time. Clinically, we have been observing increased pediatric dermatology no‐show rates and have had to find alternative methods for delivering dermatological care to this population. We aim to quantify the impact of the current events on our dermatologic clinical operations and emphasize options for providing care for vulnerable populations during political unrest.

Late cancellation and no‐show visits (LCNSV) of three University of Minnesota (UMN) pediatric dermatology clinical sites and eight UMN adult dermatology (general and surgical) clinics, stratified by suburban and metropolitan locations, were collected and analyzed for December 2024/January 2025 and December 2025/January 2026. Changes in proportions of LCNSV to total scheduled visits (LCNSV plus completed visits) were compared across years using a chi‐squared test.

Metropolitan pediatric dermatology clinical sites demonstrated a statistically significant difference in LCNSV year over year, with an increase of 5.82% (*p* < 0.001), while pediatric suburban areas showed a slight change of 1.2% (*p* = 0.64) in LCNSV which did not reach statistical significance (Table 1). Adult dermatology visits were similar across the years in LCNSV in both metropolitan and suburban settings (Table 1).

**TABLE 1:** The change in late cancellations and no‐show visits year over year for the 2‐month period of December and January stratified by adult and pediatric clinics as well as metropolitan and suburban locations.

	Completed visits	Late cancellation/no‐show visits	Absolute difference in proportion of late cancellation/no‐show visits year over year	*p* *
	No. (%)	No. (%)		
Pediatric				
Metropolitan			5.8%	*p* < 0.001
12/2024 to 1/2025	987 (79.5%)	254 (20.5%)		
12/2025 to 1/2026	1371 (73.7%)	488 (26.3%)		
Suburban			1.2%	*p* = 0.64
12/2024 to 1/2025	305 (85.7%)	51 (14.3%)		
12/2025 to 1/2026	407 (84.4%)	75 (15.6%)		
Adult				
Metropolitan			−0.7%	*p* = 0.39
12/2024 to 1/2025	3278 (85.3%)	564 (14.7%)		
12/2025 to 1/2026	2791 (86.0%)	453 (14.0%)		
Suburban			0.5%	*p* = 0.35
12/2024 to 1/2025	7246 (86.9%)	1096 (13.1%)		
12/2025 to 1/2026	7680 (86.4%)	1213 (13.6%)		

* Differences in frequency of late cancellation and no‐show visits versus completed visits year over year were evaluated using the chi‐squared test.

Previous studies have demonstrated the increased likelihood of vulnerable communities to miss or actively not attend medical appointments, regardless of medical need, during periods of social unrest [3]. Minneapolis has its highest concentration of ICE within the metropolitan areas, which parallels the location with the highest density of immigrant families [4]. Our clinical data suggest that children in metropolitan areas are disproportionately at risk of not receiving dermatological care during the surge of ICE enforcement from December 2025 to January 2026 compared to the prior year. This disruption in dermatological care is very concerning to us as clinicians. Limitations of the data include confounding variables resulting in late cancellations and no show that are not always provided or documented, and could include factors such as seasonal weather variation, insurance coverage, transportation barriers, and seasonal respiratory illness.

Teledermatology remains a critical tool that we can utilize to maintain dermatologic care for vulnerable populations [5]. We advocate for the longevity of teledermatology, as it proves once again to be a swift and powerful tool to serve vulnerable patients. We have actively increased our pediatric teledermatology visits by 225% in January 2026 (from 8 to 26 virtual visits) compared to January 2025. We also increased clinical availability at satellite/suburban locations as we have found lower no‐show and late cancellation visits in our clinical data (Table 1). Lastly, dermatologists can advocate for patients' health, encouraging care for others and doing no harm [6]. Our persistent voices in combination with skillful care, including via teledermatology, remain our strongest asset to provide continued medical care despite ongoing unrest.

## Funding

The authors have nothing to report.

## Consent

The authors have nothing to report.

## Conflicts of Interest

The authors declare no conflicts of interest.

## Data Availability

The data that support the findings of this study are available from the corresponding author upon reasonable request.

## References

[1] B. E. Trappey , “We do Care,” New England Journal of Medicine 394, no. 9 (2026), https://www.nejm.org/doi/10.1056/NEJMc2601288. 10.1056/NEJMc2601288 41744317

[2] T. T. Gelaw , M. A. Getahun , A. M. Bayih , et al., “The Impact of Civil Unrest on Child Health Care: Evidenced by Acute Medical Complications at Presentation ‐ A Retrospective Comparative Study,” PLoS One 20, no. 4 (2025): e0320902, 10.1371/journal.pone.0320902. PMC11981157 40202961

[3] S. Shimotsu , A. Roehrl , M. McCarty , et al., “Increased Likelihood of Missed Appointments (“No Shows”) for Racial/Ethnic Minorities in a Safety Net Health System,” Journal of Primary Care and Community Health 7, no. 1 (2016): 38–40, 10.1177/2150131915599980. PMC5932666 26286688

[4] Minneapolis City Council Research Department , “Profile of the Foreign‐Born Population, Minneapolis‐St. Paul,” accessed February 15, 2026, https://lims.minneapolismn.gov/Download/RCAV2/16969/Profile‐of‐Foreign‐Born‐Population‐Minneapolis‐St‐Paul.pdf.

[5] N. Chuchvara , R. Patel , R. Srivastava , C. Reilly , and B. K. Rao , “The Growth of Teledermatology: Expanding to Reach the Underserved,” Journal of the American Academy of Dermatology 82, no. 4 (2020): 1025–1033, 10.1016/j.jaad.2019.11.055. 31811880

[6] A. T. Chen and V. H. Murthy , “The Power of Physicians in Dangerous Times,” New England Journal of Medicine 392, no. 19 (2025): 1873–1875, 10.1056/NEJMp2502493. 40305692

